# Expanding Neonatal Bloodspot Screening: A Multi-Stakeholder Perspective

**DOI:** 10.3389/fped.2021.706394

**Published:** 2021-10-06

**Authors:** Tessa van Dijk, Adriana Kater, Marleen Jansen, Wybo J. Dondorp, Maartje Blom, Stephan Kemp, Mirjam Langeveld, Martina C. Cornel, Sylvia M. van der Pal, Lidewij Henneman

**Affiliations:** ^1^Department of Human Genetics and Amsterdam Reproduction and Development Research Institute, Amsterdam UMC, Vrije Universiteit Amsterdam, Amsterdam, Netherlands; ^2^Department of Health, Ethics and Society, CAPHRI Care and Public Health Research Institute, and Research School GROW for Oncology & Developmental Biology, Maastricht University, Maastricht, Netherlands; ^3^Laboratory for Pediatric Immunology, Department of Pediatrics, Willem-Alexander Children's Hospital, Leiden University Medical Center, Leiden, Netherlands; ^4^Laboratory Genetic Metabolic Diseases, Department of Clinical Chemistry, Amsterdam UMC, Amsterdam Gastroenterology and Metabolism, University of Amsterdam, Amsterdam, Netherlands; ^5^Department of Endocrinology and Metabolism, Amsterdam UMC, University of Amsterdam, Amsterdam, Netherlands; ^6^Department of Child Health, TNO, Leiden, Netherlands

**Keywords:** parental autonomy, public health, psychosocial aspects, ethics, qualitative research, neonatal screening, heel prick

## Abstract

Neonatal bloodspot screening (NBS) aims to detect treatable disorders in newborns. The number of conditions included in the screening is expanding through technological and therapeutic developments, which can result in health gain for more newborns. NBS expansion, however, also poses healthcare, ethical and societal challenges. This qualitative study explores a multi-stakeholders' perspective on current and future expansions of NBS. Semi-structured interviews were conducted with 22 Dutch professionals, including healthcare professionals, test developers and policy makers, and 17 parents of children with normal and abnormal NBS results. Addressed themes were (1) benefits and challenges of current expansion, (2) expectations regarding future developments, and (3) NBS acceptance and consent procedures. Overall, participants had a positive attitude toward NBS expansion, as long as it is aimed at detecting treatable disorders and achieving health gain. Concerns were raised regarding an increase in results of uncertain significance, diagnosing asymptomatic mothers, screening of subgroups (“males only”), finding untreatable disorders, along with increasingly complex consent procedures. Regarding the scope of future NBS expansions, two types of stakeholder perspectives emerged. Stakeholders with a “targeted-scope” perspective saw health gain for the neonate as the exclusive NBS aim. They thought pre-test information could be limited, and parents should be protected against too much options or information. Stakeholders with a “broad-scope” perspective thought the NBS aim should be formulated broader, for example, also taking (reproductive) life planning into account. They put more emphasis on individual preferences and parental autonomy. Policy-makers should engage with both perspectives when making further decisions about NBS.

## Introduction

Neonatal bloodspot screening (NBS) is offered to parents for the early detection of, mostly autosomal recessive, disorders in their child. Over the past decades, the wide international consensus has been that NBS should aim at achieving significant health gain for the child. Programs principally include disorders that pose a serious health problem for the child, where timely detection allows for effective treatment or prevention, based on the Wilson and Jungner principles for screening (1). Technological developments in screening, diagnostics and treatment have led to an ongoing expansion of the number of disorders included in these NBS programs. However, the screening principles for inclusion of conditions are interpreted and applied differently worldwide as reflected by differences in the scope of NBS between countries (2). Besides, the possibility to simultaneously test for a much wider range of disorders than those fitting within the original aim of NBS, has given new impetus to the debate about whether other benefits than health gains for the child might legitimate inclusion of disorders in NBS programs (1, 2). In 2015, the Health Council of the Netherlands advised that NBS be expanded to include an additional 14 disorders, resulting in a program targeting 31 disorders in total (3). This was based on applying the classical principles of NBS (1). Some of these 14 disorders became eligible for NBS because of the availability of new screening methods, such as measuring T-cell receptor excision circles (TRECs) for severe combined immunodeficiency (SCID) screening (4). Although the current expansion improves the early diagnosis and treatment for more disorders, there are several challenges. Screening for certain disorders that meet the screening principles, such as for SCID (see Box 1A) (4–7) may lead to unsolicited findings, due to the lack of specificity of the biomarker used. While incidental findings are to be prevented as much as possible, especially where this concerns an untreatable disorder, the question arises whether such NBS findings should be reported back to the parents (6). For other disorders, screening of subgroups, such as males only, are recommended because of great differences in disease manifestations between males and females, as is the case for X-linked adrenoleukodystrophy (ALD, see Box 1B) (3, 8–10). Finally, screening for certain disorders such as Organic Cation Transporter 2 deficiency (OCTN2 deficiency, see Box 1C) (11, 12) might detect mildly affected or asymptomatic mothers, because abnormal biomarkers in the neonate can be caused by an aberrant biochemical profile in the mother. Diagnosing mothers with a metabolic problem is outside the goal of NBS, and it is unclear what the medical and psychosocial impact is for these women.

Box 1A **Untreatable unsolicited findings: severe combined immunodeficiency (SCID).**

In the screening process of treatable disorders, nonspecific biomarkers may lead to unsolicited and untreatable findings. In case of SCID, a severe immunodeficiency presenting with severe infections during the first months of life, early detection is crucial because children do not survive the first year of life without treatment (4). However, the biomarker used in SCID screening (T-cell receptor excision circles; TRECs) is nonspecific and might also indicate other disorders with T-cell lymphocytopenia for which no treatment exists, such as Ataxia Telangiectasia (5, 6). It is unclear whether these should be included and reported. The challenges of SCID are studied in a Dutch pilot study, the SONNET-study, on the implementation of SCID in the Dutch NBS program (4, 6).

Box 1B **Screening subgroups: X-linked adrenoleukodystrophy (ALD).**

X-linked disorders may pose a challenge to the aim of NBS, since these can be severe in males, while female carriers are often mildly affected or asymptomatic. Although health consequences for these females themselves may remain limited, carrier ship may have consequences for families and future reproductive decisions. This raises the question whether it is desirable to screen females for X-linked disorders in NBS. In the case of ALD, male patients can develop adrenal insufficiency and demyelinating lesions in the cerebral white matter for which early diagnosis is essential to initiate effective treatment (8). Females only very rarely (<1%) develop adrenal or cerebral disease manifestations, but 80% develops a myelopathy during late adulthood (9). There is currently no treatment for ALD-related myelopathy and neuropathy. The Dutch Health Council therefore advised that NBS screening for ALD should detect only affected males (3). It is currently unknown how screening of subgroups in NBS is perceived by the general public. The SCAN study aims to identify the clinical and laboratory aspects of this subgroup screening in a pilot study on the implementation of ALD in the Dutch NBS programme (10).

Box 1C **Identifying asymptomatic mothers: Organic Cation Transporter 2 (OCTN2) deficiency.**

In some cases NBS may lead to the detection of a disorder in the mother rather than the neonate (11). OCTN2 deficiency for example, is identifiable in the Dutch newborn screening (NBS) program through a low carnitine level in the acyl carnitine profile in dried blood spot. However, this finding might also indicate OCTN2 deficiency in the mother, instead of the neonate. It is currently unknown if these, often asymptomatic, mothers are at risk for OCTN2 deficiency-related complications, such as cardiomyopathy and arrhythmias, and whether follow-up and/or treatment is required (11, 12). These women are confronted with a medical diagnosis of their own during their maternity period and the psychosocial impact of this is currently unknown. Recently, it was decided in New Zealand to discontinue newborn screening for OCTN2 deficiency (12).

The questions complicating the inclusion of these disorders (SCID, ALD, and OCTN2) can be regarded as exemplary issues that are associated with expanding NBS on the basis of current screening principles. Similar issues can be expected to emerge with the possible future inclusion of disorders, especially those outside the original NBS aim. A specific concern is that the presently high professional and the public support for NBS may be weakened when benefits of screening are perceived as less evident. It is therefore important to explore the perspectives and views of different stakeholders in these matters. Research has shown that parents often express a positive attitude toward expanded NBS (13–15). Professionals' opinions, however, may diverge from this viewpoint as they often put more emphasis on the possible harms of expanded screening (16, 17). Discussions on the aim and scope of NBS do not only relate to the type of disorders that are screened for, but also to the information provision for parents and consent procedures (18). For an in-depth exploration on how different stakeholders, both parents and professionals, value the current NBS expansion, as well as their views and expectations regarding future developments, we conducted a qualitative interview study in order to answer the question how different stakeholders think about NBS and its expansion. In an era of changing and expanding screening programs it is important to conduct studies on stakeholder perspectives to be able to reflect on the extent to which the offered program continues to meet users' acceptance and needs. In 2021, the Dutch screening program has been expanded to 25 disorders. The expansion is still ongoing with many challenges still ahead. In parallel with this study on psychosocial aspects, several pilot studies have started, e.g., on the implementation and decision making regarding SCID, ALD, and OCTN2 (10, 19). This is the first study in the Netherlands on the stakeholder perspectives toward the NBS program and the challenges regarding its expansion. It is part of a bigger study project on NBS and its expansion, which also includes a quantitative survey study on parental opinions and experiences with NBS. This study aims to fuel public debate and contribute to future policy decisions and shaping NBS practices.

## Methods

A qualitative research method was chosen to explore the range of stakeholders' perceptions and expectations. An interview allows probing questions and explore underlying reasoning of participants. The Medical Ethical Committee of the VU University Medical Center Amsterdam approved the study protocol (no. 2019-509).

### Participants and Procedure

**Figure 1:**
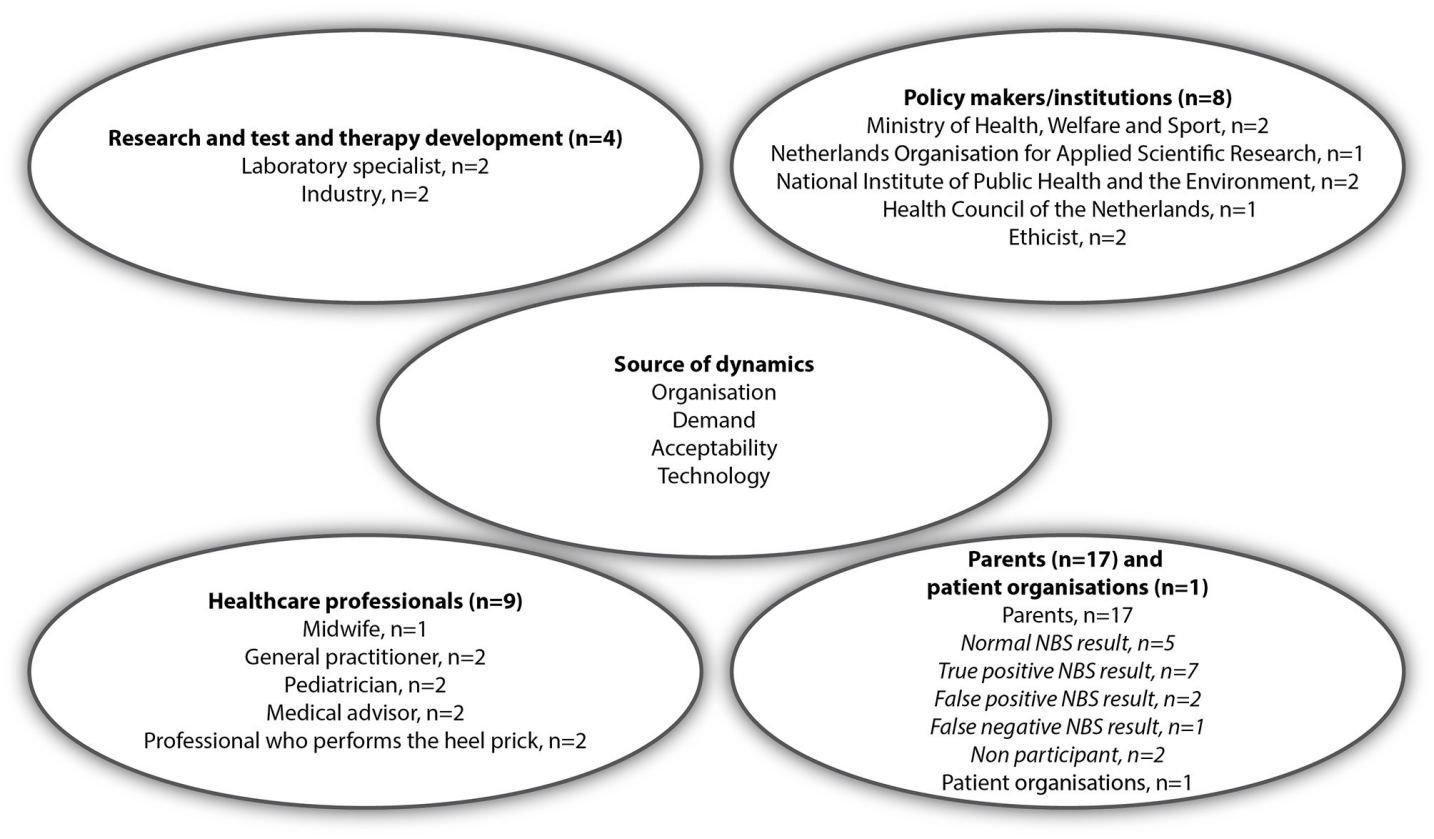
Interviewees categorized by the four different stakeholder groups in the Network of Actors model [adapted from Achterbergh et al. (20)].

To identify relevant groups of stakeholders, we used a Network of Actors model in which stakeholders from different fields need to enter a process of mutual learning and get attuned for implementation and acceptance of new technologies in society and healthcare (20). Based on this model we included four main groups: healthcare professionals involved in NBS, institutions involved in NBS policy making, researchers and laboratory specialists involved in test and therapy development, and parents and patient organizations (Figure 1). In the Netherlands the newborn screening program is a public program which is organized and conducted by the Dutch National Institute for Public Health and the Environment (RIVM). The RIVM provides the information for parents, coordinates the screening in the five screening laboratories and provides abnormal test results to general practitioners (GPs). GPs inform the parents and refer them to the hospital for diagnostic conformation and follow-up care. Information on NBS, including a leaflet, is provided during pregnancy by an obstetric healthcare provider, and provided before the newborn blood spot is collected at home by a youth health care worker, maternity nurse or midwife. When the baby is admitted to the hospital during the first week after birth, the blood spot is collected by a hospital health care worker. Regarding consent, parents are asked if they agree to NBS. NBS is not mandatory, though few parents decline screening. Professional stakeholders, all involved in NBS, were recruited for the interviews using purposeful sampling, based on their role, experiences or expertise. In some cases, such as policy makers, they were recruited by snowball sampling. Stakeholders were sent an invitation including an information letter by email. Four professionals refrained from participation. In two cases, professionals preferred a joint interview together with a direct colleague. In total, 22 professionals were interviewed after which data saturation was reached. Five interviews were conducted by two interviewers (AK and TD) and fifteen by a single interviewer (AK or TD). Parents were also included in the study, in order to learn their demands and acceptability of a (future) expanded NBS program, even when they would not face abnormal NBS results themselves (20). Parents were recruited via three patient associations platforms, social media, and a pediatrician. No specific eligibility criteria for inclusion of parents were formulated, other than that they were offered NBS maximum 2 years before participation. In total, 17 parents were interviewed including parents of children with a normal (*n* = 5), a false-positive (*n* = 2), a false-negative (*n* = 1) or a true-positive (*n* = 7) NBS result and parents who declined NBS for their child (*n* = 2) (Figure 1). The mean age of the group of parents was 33.2 (range: 27–40) and a majority had a high education level (77.8%) (Supplementary Table 1). Interviews were conducted face-to-face, by telephone, or video call. All interviews were conducted between October 2019 and April 2020, and lasted between 30 and 90 min. Written informed consent was obtained from all participants.

### Interview Guides

Based on the literature, and in collaboration with a multidisciplinary team, two separate semi-structured interview guides were developed for professionals and parents. The interview guide addressed the current and future expansions of the Dutch NBS program and were based on Goldenberg et al. (21), who identified key ethical, legal and social implications (ELSI) of NBS. Topics applicable to the Dutch situation and the intended expansion including the condition-specific issues were selected and formed the starting point of the interview guide. The interview guides for professionals and parents (Supplementary Appendices A,B) addressed their involvement in and experience with NBS, respectively, and their opinions and perspectives on practical, ethical and psychosocial aspects of NBS developments and expansions.

### Data Preparation and Analysis

The interviews were audio-recorded and transcribed verbatim. We complied with the 32-item COREQ checklist for reporting and analyzing the data (22). Thematic analysis (23) was done in parallel with interviewing. Using Atlas.ti software, interviews were coded using inductive coding, by two researchers independently (AK and TD) to increase reliability. Codes were grouped and ranked into a code tree based on the main themes that emerged from the data (Supplementary Appendices C,D). Results were discussed with AK, TD, and LH. These researchers are trained in and experienced with qualitative research. AK and TD generated a code tree independently, for both the interviews with parents and professionals, and after three interviews compared the way of coding. Based on these two concept code trees a definitive code tree was developed for the analysis of the next interviews. After six interviews, again the way of coding was compared and discussed. Differences in coding and findings were discussed until consensus was reached. Quotes used in the manuscript to illustrate themes were translated from Dutch.

## Results

Three main themes emerged from the data and will be discussed accordingly: (1) Benefits and challenges of the current expansion of NBS, (2) Future expansions: new developments and broadening the aim of NBS, and (3) NBS acceptance and consent procedures.

### Theme 1: Current Expansion of NBS: Benefits and Challenges

Both parents and professionals expressed a positive attitude toward the current NBS expansion as advised by the Health Council of the Netherlands (3) indicating health gain for the newborn as the most important benefit. Many stakeholders underlined treatability and health gain as a prerequisite for inclusion of a disorder in the NBS program. As long as screening is aimed at the early detection of treatable disorders, as is the case with the current expansion, stakeholders generally did not have any objections.

“*Yes, I would say: throw everything into the test that you can, whatever meets the criteria, whatever that will help you, of course. Yes, that is fantastic, then you would have more healthy children.”* [Parent 6, normal NBS result]

“*That treatment can be offered to more children, resulting in health gains. That, I think, is goal number one.”* [Professional 15, policy maker]

Professionals believed that the Dutch screening program is well-organized, with solid infrastructure and social acceptance, all of which provide a solid foundation for expansion(s) at a reasonable cost

“*But, you already have that infrastructure in place, right, of professionals who draw […] blood. Logistics with the entire heel prick card, with laboratories. […] Together, […] you have organized social legitimacy and support. Then you actually want to use it as efficiently as possible. […] In principle, you can actually achieve even more health gains at an acceptable cost.”* [Professional 15, policy maker]

Despite the health gain for neonates, stakeholders also saw several challenges to the current NBS expansion and mentioned aspects that should be considered critically when expanding further, which are discussed below.

#### Uncertain Results: Weighing the Psychological Impact

Stakeholders indicated that an increase in the number of disorders included in NBS could lead to an increase in false positive results or in results of unknown clinical significance. They mentioned that the full phenotypical spectrum of many disorders, also in the current NBS, is still unknown and that in some cases screening could lead to the detection of mild disease variants. In this situation it is unclear whether the newborn must be treated as a patient or not. Stakeholders thought this would create much anxiety and uncertainty for parents which should be taken into account when thinking about further expansions.

“*So, that means that such an expansion of such a heel prick will help a lot of patients. But it could be that you get a lot of false positive results, or results where you don't know the exact meaning. And I think the latter group is the most problematic because, in my opinion, what is very troublesome for parents is uncertainty.”* [Professional 19, pediatrician]

Two parents, whose newborn received a diagnosis of a mild disorder struggled with uncertainties regarding prognosis. Although they were positive about expansion of NBS they indicated that they would rather not have known the diagnosis because of a lack of symptoms in the child and uncertain clinical relevance and were hesitant to include these kind of disorders in a NBS program.

“*I found it very difficult to accept that she [my child] has this [diagnosis], I found that very difficult to grasp. […] Yes, I would rather have not known in that respect, also, because I think: she did very well. Only, yes, there also are other children, of course, where that is not the case.”* [Parent 12, positive NBS result, mild phenotype]

“*They just literally say that: 'We don't know what it does [the identified condition]. What we do is all preventive.' And then I think: but then you should not include it in the heel prick. So that's what I keep running into all the time.”* [Parent 9, positive NBS result, mild phenotype]

One mother who obtained a false positive result acknowledged the psychological consequences of uncertain results, but thought these consequences do not outweigh the benefit of health gain for newborns.

#### Unsolicited Untreatable Findings: To Report or Not to Report?

Many parents indicated that they would want to be informed about any disorder detected through NBS, regardless of whether there are treatment options available. Some parents saw it as a parental right to receive any information about their child, with a corresponding duty on the part of healthcare professionals not to “withhold” such information once it was known.

“*If it is something that is not visible, looking at the child, but being the caregiver you know it, and you would not say anything to the parents, then I think you are withholding information. Yes, I think you have an obligation to tell, no matter how troublesome that news is.”* [Parent 2, normal NBS result]

Apart from parents' appreciation for receiving detailed medical information regarding their child, parents thought that an unsolicited finding that involves an early diagnosis of an untreatable condition could be beneficial, as it would save them medical visits and tests in search of a diagnosis, and reduce the period of uncertainty after the onset of the first symptoms in their child. In addition, parents stated that they preferred to learn about a diagnosis of an untreatable condition at an early stage, giving them time to prepare and anticipate symptoms in their child. It was reasoned that the grief that comes with a diagnosis would occur regardless of the timing.

“*First, you get trapped in endless visits from hospital to hospital, perhaps. Yes, and only then will you find out [what disease it is].”* [Parent 7, positive NBS result]

“*Yes, that then [in the case of an untreatable disease] you can still be alert to signals that something is wrong, you can safely do something that might postpone or stabilize it or […] prevent things from happening.”* [Parent 13, positive NBS result]

“*So yes, the grief will come anyway, won't it, whether it is sooner or later. If the disease starts to manifest itself, then the grief is there, too.”* [Parent 2, normal NBS result]

One parent and a few professionals expected that some parents might want to know about untreatable disorders because it may influence their decision about a future pregnancy. These professionals however, were very reluctant about using NBS for reproductive decisions. Reporting or not reporting untreatable unsolicited findings was often placed in a broader discussion regarding the general aim of NBS and the question whether NBS should be used to screen for untreatable disorders.

“*I don't think we should screen for untreatable conditions a priori. […] But if these are identified as incidental findings […], in my opinion, that is acceptable as collateral damage.”* [Professional 6, medical advisor]

“*Well, that is the problem of non-treatability, and what do you do with that information? I'm not quite sure.”* [Professional 14, ethicist]

#### Screening Subgroups: Justified by the Goal of NBS

Both professionals and parents acknowledged that selective testing of males only for ALD is a dilemma, giving the arguments in favor of and against ALD screening in girls. They agreed with the current Dutch policy not to screen females (Box 1B) because of the aim of NBS: testing for treatable disorders for health gain. Most parents did not seem to perceive the screening of subgroups as problematic in itself.

“*I can understand that. I don't see that [not screening girls for ALD] as a discriminator, I see that as something with a medical reasoning. As I just said, if a certain clinical picture only occurs when you are considerably older, yes, then the question is whether you want to know. And that child, I assume, can decide later whether she wants to know. And if it is not treatable after all, then I think: yes, what's the point?”* [Parent 6, normal NBS result]

However, several parents indicated that they would prefer to be informed if their daughter was found to have ALD because this might help to anticipate possible symptoms. Some felt uneasiness that the opportunity to learn about ALD in girls is not included in the NBS program. These feelings partly related to a perceived lack of autonomy, with others deciding for them that their daughters are not to be screened for ALD.

“*Well, in any case, it is right that it does involve boys. But it really seems very strange to me. […] Well, that you could already know something by, yes, something quite easily, but then it is decided for you by the RIVM or by someone else that you don't get that information.”* [Parent 14, positive NBS result]

Contrarily, other stakeholders especially emphasized the child's right not to know, because the knowledge of a late-onset untreatable disorder might be experienced as burdensome.

“*Yes, but it must be treatable. That's what makes that question so difficult, because if you can't treat it, but you have the diagnosis, then what's the point of that? […] You can't do anything with it.”* [Parent 4, normal NBS result]

These parents and professionals also emphasized the value of living years without this knowledge, the so-called “golden years.” Furthermore, stakeholders thought that the NBS information for parents should not give too much emphasis to the fact that girls are not tested, because it could raise questions and concerns among parents. But others thought that it should be mentioned that girls are not tested, otherwise parents might perceive a diagnosis of ALD in later life as a missed diagnosis which could reduce their confidence in NBS.

“*Many parents just assume that if you come out clear from the heel prick, then you are just healthy. And I think it will really surprise people […] if there is a child with PKU who was not identified by the heel prick, they will think: the heel prick is wrong. And, so there will soon be girls with ALD who will not be picked up by the heel prick, yes, unless the policy changes.”* [Professional 12, policy advisor]

#### Identifying Mild Disease Phenotypes in Mothers

When introducing the challenges regarding the diagnosis of OCTN2-deficiency in asymptomatic mothers, many parents asked for more detailed information regarding the severity, prognosis and possible treatment options. Despite the uncertain clinical significance of OCTN2 deficiency in asymptomatic adults, parents indicated several benefits such as the possibility to make lifestyle adaptations, to be alert for possible symptoms, and to avoid a long search for the correct diagnosis in case symptoms would appear. However, parents also stated that they did not want to be burdened with information on their own health, especially if the clinical consequences are unclear. Some parents also thought that the maternity period is a bad moment to receive this information. Overall, parents were not very outspoken on this topic. Professionals were more outspoken about diagnosing OCTN2 in asymptomatic mothers with NBS. They felt uncomfortable with imposing a diagnosis and regular health checks on people without health complaints, especially since these women may remain asymptomatic all their lives. It was also stated that the detection of disorders in women was beyond the scope of NBS. They thought this might compromise the straightforward aim of the screening program and complicate communication with parents.

“*Do you have to screen for OCTN2? That is one that may clearly be reconsidered, because what are the disadvantages for the program as a whole. […] It does not contribute to a clear program. […] It's really a bit disruptive, I think.”* [Professional 12, policy advisor]

“*And we know as well, about the OCTN2, […]: yes, sometimes you identify a mother who is affected but had not been bothered by it until she was 30 and you will tell her: 'You are not completely healthy and we are going to keep an eye on that.' Yes, then something will change in her life.”* [Professional 2, medical advisor]

### Theme 2: Future Expansions: New Developments and Broadening the Aim of NBS

Two topics were explored regarding future developments and expansion of NBS: new test and treatment options, and broadening NBS's aim. Firstly, professionals stated that NBS expansion will be driven by the development of (gene-) therapies for rare disorders, as was recently the case with spinal muscular atrophy.

“*At the moment, there is a lot of research relating to treatments for many disorders that cannot be treated today. So, I think that […] this will also drive implementation of more diseases to be included in the newborn screening. Then, of course, we also see development—going from doing biochemical testing to moving more and more into DNA-testing. At the moment, it's quite expensive, so there is a need for more cost-efficient solutions in genotype screening before that will be implemented wide scale. But of course, the trend is in that direction”* [Professional 17, industry]

Most professionals expected that next-generation sequencing (NGS) might increasingly be applied in NBS, not only as a follow-up diagnostic test, but also as a first-tier test for some categories of disorders. However, professionals thought that the interpretation of genetic variants needs be improved, to avoid results of uncertain significance. In addition, methods must be found to avoid an overload of unsolicited findings and reduce the current costs of NGS, which are much higher than metabolite screening and more targeted DNA-testing. Finally, some disorders screened for in NBS are not (always) genetic, such as congenital hypothyroidism, which means NGS will not be able to completely replace metabolite screening in NBS. Most parents were not critically concerned with using NGS and thought that the NBS practice would not differ that much, provided that the privacy of DNA data was guaranteed.

Secondly, several parents saw advantages of a broader aim of NBS, such as knowing about untreatable disorders and preventing associated diagnostic delays, but also saw disadvantages such as the loss of carefree time with a child.

“*So, if you have it [untreatable conditions] in the heel prick screening, you prevent that quest [for a diagnosis]. On the other hand, the time with your baby is no longer so carefree, because then you already know that the child has a serious illness.”* [Parent 5, Normal NBS result]

Professionals acknowledged parents' and patient advocacy groups' wish to be informed about all NBS results and obtain as much information on their child's health as possible. However, several professionals disagreed with screening for disorders currently marked as lacking effective interventions, indicating the tension between the goals of NBS in the domain of public healthcare and the preferences of some individuals.

“*But it's about the group, that's the problem. The screening is set up for the group and not for one child.”* [Professional 12, policy advisor]

According to some professionals even if a majority of parents would choose to be informed about an untreatable disorder in their child, this should not be the general rule because it denies a “right not to know” to a minority of parents. They felt that this right should be protected.

“*Putting democracy over ethics can be quite dangerous sometimes, of course. […] since, you know, ethics is not always democratic, and should be aimed at protecting minorities, perhaps.”* [Professional 8, ethicist]

Related to that, some parents feared that in case of knowing about late-onset untreatable disorders children's chances and possibilities will be limited, and therefore children should not be burdened by knowing about these disorders. Parental advantages of screening for untreatable disorders, e.g., informing future reproductive decision-making, were often considered as “secondary” and insufficient justification for future inclusion in the screening program. In contrast, some professionals had a broader view on the concept of health gain for the child and thought that other advantages of an early diagnosis in newborns should be considered, including improving the quality of life and shortening of diagnostic delays or uncertainty. One professional thought that unsolicited findings should be reported to parents if they request this information, even if this is considered an untreatable disorder, as parental wishes are sometimes unfairly pushed aside or overruled by ethicists with little understanding or knowledge of diseases.

“*I think that [NBS's goal] is a bit old-fashioned. […] I do have my doubts about how twisted everything is to prevent incidental findings, for example. […] I see, of course, a subset of parents, but they say, ‘Oh, please, if we had only known from the beginning, we wouldn't have had all that hassle until diagnosis.’ […] Just tell them, then they can at least move on with it, because the idea that you do people a lot of injustice if you tell them, I don't think that's true at all.”* [Professional 4, paediatrician]

Stakeholders with a broader concept of health gain often saw room for a broader application of NBS or a second heel prick for untreatable disorders, which challenges the current strict interpretation of the traditional screening principles which require availability of a treatment (1). However, a few professionals mentioned possible financial consequences of including very rare disorders with very expensive treatment options for society.

“*How much [money] are we going to spend on treatments for severe disorders that are very expensive? […] the treatments that are possible should not necessarily be given. There are limits and we construct these as society.”* [Professional 14, ethicist]

### Theme 3: Acceptance and Consent Procedures of (Expanded) NBS

Many stakeholders saw a connection between defining health gain for the neonate as the clear and uniform goal of NBS and the high societal acceptance of NBS.

”*Because of the health gain, for example, the Wilson and Jungner criteria are actually the foundation of such a program, to sell it to the public as well."* [Professional 1, patient organization]

In addition, that the program is solely for the benefit of the child was also seen as a “justification” of the current concise consent procedure: many stakeholders thought it is sufficient to only briefly explain the purpose of NBS and to be somewhat directive in offering screening.

“*And one of the choices [is]: we want a transparent program. If you are called after the heel prick, then we can make a difference for your child. […] You have the opportunity to say 'no', but you are supposed to say 'yes'. And that is justifiable from an ethical point of view.”* [Professional 12, policy advisor]

Stakeholders, in general, agreed with the current concise consent procedure, although it was mentioned that not all parents received or read the information and that the consent procedure could be improved. Parents indicated that they prefer verbal information provision.

One professional argued that more detailed information about the disorders is essential to maintain high uptake of NBS.

“*You can't say, 'Well, that disease, that's way too complicated for people' and expect everyone to continue to fully participate in a heel prick program involving 31 metabolic diseases.”* [Professional 1, patient organization]

When screening would include untreatable disorders, according to several stakeholders the informed consent procedure should be more detailed and personalized so that parents have the possibility to decide for themselves about untreatable (unsolicited) findings.

“*Screening for untreatable conditions, that requires a considerable amount of counselling to get parents to realize: do I want to participate or not? Do I want to know in the first weeks after birth that my child will develop a disease at a later age and will require care?”* [Professional 15, policy maker]

They drew a parallel with non-invasive prenatal testing (NIPT) for fetal aneuploidy in the Netherlands, where parents can decide whether they want to be informed about unsolicited findings, i.e., chromosomal abnormalities other than the common trisomies.

“*Well, anyway you shouldn't inform all parents by default [regarding untreatable conditions]. You either have to ask, and they have to actively give permission, just like the NIPT […].”* [Parent 5, Normal NBS result]

Others thought adding “choice options” in NBS would put too much pressure on the parents and complicate parental decision-making.

“*Well… [untreatable disorders], that creates the risk that you make it too complicated for people, and also that you might make people feel guilty if later on it turns out to be a problem and they think: ‘if only I had ticked that box’. I am afraid of that.”* [Professional 19, pediatrician]

Some stakeholders thought that parents should be protected against burdensome choices.

“*But, I think that if you do start screening conditions that are not treatable…, I think it will be disappointing how many people really want to know. I think a lot of people say that they want to know all kinds of things, but in fact we have to protect them a little from themselves.”* [Parent 3, normal NBS result]

Furthermore, parents mentioned the balance between complete information and an unnecessary overload of it, as they expected that parents could not process a lot of the information. Professionals mentioned the balance between on the one hand providing complete information and on the other hand not creating anxiety because this might lower the NBS uptake.

“*You don't want to worry parents unnecessarily. You also want to keep the participation rate high. You don't want everyone to say, 'I don't want that' after reading the brochure, while you do want to be honest: well, that's what we looked for.”* [Professional 2, medical advisor]

In addition, it was mentioned that extensive counseling of all parents for individual decision-making about testing for very rare findings is not feasible in practice.

“*Actually, [information about detecting untreatable conditions] should be in the brochure, in any case. Look, if we have to tell them all that, then it will become so medically substantive, we are not trained for that. But I do think parents have a right to know.”* [Professional 11, professional who carries out the screening]

## Discussion

This study aimed to identify stakeholders' perceptions, opinions and expectations regarding current and future NBS expansions. All stakeholders highly valued the primary aim of achieving health gain for newborns. Stakeholders identified several challenges to the current NBS expansion and mentioned aspects that should be considered critically when expanding. Thematic synthesis of the study findings revealed that roughly two distinct types of perspectives could be identified among both professionals and parents on NBS expansion and its scope. These views differed on the definition of health gain, how parental and societal benefits of NBS should be weighted, on the amount of pre-test information and the consideration of parental autonomy (summarized in Table 1). Stakeholders at the one end of the spectrum reasoned mainly from a public health perspective, which in their opinion meant that NBS should be targeted at achieving health gain for the population. Therefore, screening should be aimed at treatable disorders only, and the only *primary* beneficiary of NBS is the newborn. Relatives of the newborn may benefit from an early diagnosis as well, for example parents can base reproductive choices on this knowledge, but these are considered *secondary* goals, seen from this perspective. Screening only for disorders for which treatment is available is important to safeguard the clarity of the program. With a clear goal of NBS, namely early detection of disorders for which early treatment improves the clinical outcome, information provision for parents can be straightforward. Information is provided to parents in order to achieve informed participation in NBS, and does not comprise detailed information or choice options other than to participate or not. Too much information or choice options are considered burdensome according to stakeholders supporting this perspective, which we describe here as a “targeted-scope” perspective. On the other end of the spectrum are the stakeholders with what we describe as a “broad-scope” perspective. Stakeholders with a broad(er)-scope perspective were more focussed on individual preferences and personal values and less on a public health perspective. They thought that parents should be able to decide in accordance to their own values what kind of information they want to receive from the NBS in their child. Often, health gain was defined broader than in the strict medical sense, also including for example the anticipation to future health problems and reproductive planning. In contrast with the stakeholders with a targeted scope perspective, stakeholders with a broad-scope perspective tended to add more value to this type of “indirect” or secondary health gain. They also often were open to the idea of a tailored of customized NBS in which parents could decide on several conditions whether they prefer to receive this information on their newborn or not. Pre-test information should be more extensive and detailed, and tend more toward counseling were the pros and cons of testing are carefully weighted. In their perception, providing detailed information and choice options is not seen as burdensome but rather as a way to increase parental autonomy. These two types represent a simplification of the true complexity of the views expressed by the stakeholders and can be seen as the two ends of a spectrum, presented as such for the sake of clarity (Table 1). The quotes illustrate how stakeholders could position themselves in that spectrum. This synthesis might help professional stakeholders to make their starting points and priorities concrete in developing the NBS program.

**Table 1:** Two types of stakeholders' perspectives on the scope of NBS: a targeted-scope vs. a broad-scope perspective.

**Targeted-scope**	** 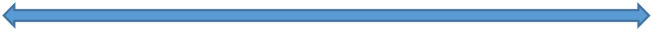 **	**Broad-scope**
• Focus on public health perspective • Narrow definition of NBS screening goals • Parents should be protected from too much information and difficult decisions	Type of scope perspective 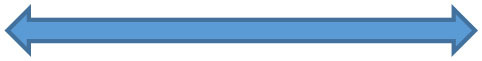	• Focus on individual preferences and personal values • Broad definition of NBS screening goals • Parental autonomy
Health gain for the newborn by screening for treatable disorders	Aim of NBS 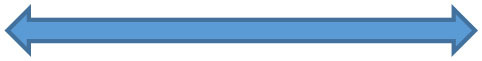	Health gain is broadly defined and also includes anticipation on future health problems and reproductive planning
Newborn	Beneficiary 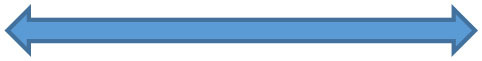	Newborn, parents, and extended family
Essential information on the goal of screening in order to achieve informed participation	Pre-test information on NBS 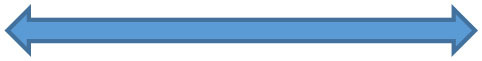	Detailed information on testing and possible outcomes in order to achieve informed choice
Limited choice options, parents can decide if they participate in the program or not	Number of choice options and autonomy 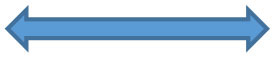	Several choice options, based on individual preferences and values

*NBS, Neonatal Bloodspot Screening*.

Earlier studies have shown that in general, parents express a positive attitude to expanded NBS while professionals are often more critical (13–17). Parents that participated in our study however, often seemed to be aware of possible drawbacks of expanding NBS, and did not necessarily fit with a broad scope perspective. On the other hand, several professionals did not coincide with the targeted-scope perspective because they thought for example that tailored NBS would be desirable in the future.

### Challenges of NBS' Current and Future Expansions

Stakeholders' support for the expansion of NBS within the aim of early detection of treatable disorders corresponds to previous studies on NBS expansion where parents and professionals also primarily emphasized the interest of the child (24, 25). In the current study, stakeholders reiterated concerns mentioned in earlier ethical analyses (21, 26) and empirical qualitative studies (13, 14) and revealed different opinions about the related challenges including uncertain results, screening of subgroups and diagnosing asymptomatic mothers. Some professionals and parents worried that these challenges might affect the support of the NBS program, because it could make the clinical utility and reliability of NBS less clear, and cause parental anxiety. Long-term psychological effects of false-positive results have been extensively studied, with divergent findings regarding the presence of long-term effects (27, 28). Some studies reported that timely information provision about the possibility of false positive results could reduce parents' fear and anxiety after hearing these results (28). This was also the experience of parents participating in this study who experienced a false positive result or obtained a diagnosis of a mild variant of a disorder from NBS.

In accordance with the literature (6, 13, 15), most parents in our study would like to be informed about unsolicited findings including untreatable disorders, because they expect that having this information would prevent a search for a diagnosis or enable them to better plan their lives. In contrast, as was previously acknowledged (2), several parents and professionals believed that reporting untreatable unsolicited findings does not fit with the NBS aim of health gain. The discussions about untreatable unsolicited findings and adding untreatable disorders to the program merged and revealed the recurring debate between the public health approach of NBS vs. and the individualized approach and parental autonomy. This discussion connects to a broader discussion about the aim of NBS and benefits other than health gain e.g., obtaining information about carrier status and late-onset disorders (2).

Most stakeholders did not perceive screening only males, in the case of ALD, as problematic in itself. Although some parents felt uneasy about the fact that girls are not screened, because ALD might have medical and reproductive consequences for them at a later age. It seems that in several states in the United States, where ALD is screened through NBS in both males and females, the detection of asymptomatic females is not perceived as a disadvantage or dilemma (29). In a study amongst ALD families, a vast majority was in favor of the inclusion of both males and females ALD screening through NBS (30). However, since NBS for X-linked disorders is relatively new, the long-term psychosocial effects of early detection for these females needs to be studied further. Parents thought that the disadvantages of detecting asymptomatic mothers with metabolic problems through NBS, such as with OCTN2 with its uncertain clinical relevance, might be outweighed by the potentially lifesaving benefit for detected newborns. This contrasts with the recent decision made in New Zealand to discontinue screening for OCTN2. In New Zealand it was found that the screening test had a poor sensitivity and poor positive predictive value. It appeared that the majority of positive screening results were due to the presence of the condition in asymptomatic mothers (12). The psychosocial impact on mothers diagnosed with a disorder through NBS is unknown, and would be relevant for decisions about the inclusion of these disorders in a national NBS program. Financial consequences were mentioned by a few stakeholders as a possible challenge for NBS expansion. Resources could potentially come at the expense of other health care sectors (18). To develop a full oversight of stakeholders opinions also the general public should be included and the impact on health costs should be discussed.

Parents differed about the preferences regarding information about untreatable conditions. In this discussion some said that children should be protected against information about their health status which might be burdensome, because no treatment is available. In case of including disorders for which it is not clear what treatment options are available and effective medical professionals and policy makers should critically reflect on this and make clear what their priorities are.

#### Informed Consent Procedures

Although a concise consent procedure was justified according to the stakeholders, some saw room for improvement since not all parents received or read the information. In literature, an improvement was also advised because parents are often not aware of the possibility of an unfavorable outcome, including false positive results (31, 32). More research could provide insight in whether more verbal information provision, as preferred by parents, about NBS might improve informed decision-making. Stakeholders thought that information provision should be improved and extended if NBS is expanded to include untreatable or late-onset disorders. However, an extensive counseling procedure might complicate the decision-making process (31), an issue also raised by stakeholders in our study, in addition to challenges regarding parental trust in and acceptance of NBS. In line with published data (14), stakeholders argued that a directive approach in offering NBS may no longer be justified because of the variety in the severity and treatability of the disorders included. Our study confirms earlier findings and is highly relevant in the light of further NBS expansions. Furthermore, it was suggested that an optional NBS test could be offered in addition to the standard NBS, similar to the choice regarding unsolicited findings in NIPT in the Netherlands. This was for example done in Wales for Duchenne muscular dystrophy, for which parents themselves had to send a separate NBS sample card back to the laboratory (33).

#### Strengths and Limitations

A wide range of Dutch stakeholders was included, which provided a broad overview of the different interests, opinions and challenges in NBS, using key ELSI questions from literature. It is possible, however, that the study included participants that were overly positive about NBS. The recruitment method of parents might have attracted individuals who were interested in the topic, which may have introduced bias. In addition, the group of participating parents was highly educated, which might have influenced the results. The sample does not include single parents and consisted mainly of women. Furthermore, the general public and hospital managers, were not involved as stakeholder in the interviews but might have different perspectives. This needs to be studied further.

## Conclusions

The ongoing expansion of the Dutch NBS program seems to be supported by the different groups of stakeholders, especially as it follows the rationale of health gain for the newborn. Regarding the scope of NBS, two types of perspectives emerged among stakeholders: a “targeted-scope” vs. “broad scope” view regarding health gain and individual parental autonomy. Policy-makers should engage with both these perspectives when making further decisions about NBS. In order to maximize support from professionals, parents and society for the program, it is important that stakeholders with different views at least find themselves heard.

## Data Availability Statement

The datasets presented in this article are not readily available because privacy regulations. Requests to access the datasets should be directed to Prof. Lidewij Henneman, l.henneman@amsterdamumc.nl.

## Ethics Statement

The studies involving human participants were reviewed and approved by Medical Ethical Committee of the VU University Medical Center Amsterdam (no. 2019-509). The participants provided their written informed consent to participate in this study.

## Author Contributions

LH and SP designed the study. TD and AK performed the interviews and analyzed the data. TD, AK, and LH wrote the paper with input from MJ, WD, MB, SK, ML, MC, and SP. All authors contributed to the article and approved the submitted version.

## Funding

This study was financially supported by the Netherlands Organization for Health Research and Development ZonMw (PANDA study, Grant no. 543002006).

## Conflict of Interest

The authors declare that the research was conducted in the absence of any commercial or financial relationships that could be construed as a potential conflict of interest.

## Publisher's Note

All claims expressed in this article are solely those of the authors and do not necessarily represent those of their affiliated organizations, or those of the publisher, the editors and the reviewers. Any product that may be evaluated in this article, or claim that may be made by its manufacturer, is not guaranteed or endorsed by the publisher.
