# Supplementary material for: Expanding Neonatal Bloodspot Screening: A Multi-Stakeholder Perspective
Source: Front Pediatr. 2021 Oct 6;9:706394. doi: 10.3389/fped.2021.706394 (PMC8527172; doi:10.3389/fped.2021.706394)
Supplement: Supplementary file 1 [file Data_Sheet_1.docx]

**Supplementary file: *Expanding neonatal bloodspot screening: a multi-stakeholder perspective***

**Supplementary Appendix A. Interview Guide: Professionals**

**1. Role in care and involvement in the expansion of the heel prick test (short)**

- What is your position and what is your professional involvement with heel prick screening?

- What do you think works well in the heel prick screening and what could be improved? How?

**2. First response to the expansion of the heel prick test / Health Council advice (2015)(3)**

- What was your first reaction to the advice to expand the heel prick test? Can you clarify this?

**3. Perspective on ethical issues related to (expansion of) the heel prick test and future outlook**

- What do you see as important advantages of the current expansion of the heel prick test?

- What do you see as significant disadvantages of the current expansion of the heel prick test?

- How do you view the disorder-specific requirements and associated questions? (*ALD*, *SCID*, *OCTN2*)

- How do you see the future of heel prick screening in general?

- What type of disorders are, according to you, suitable for inclusion in the heel prick test?
- (*Untreatable disorders? Late-onset disorders?*)
- Where is the limit? Why?

- What do you think about the option for parents to choose a 'plus package' test that includes untreatable disorders?

**4. Influence on participation, trust and provision of information**

- Do you think the current expansion or future expansions will affect the willingness of parents to participate in the heel prick test? How and why? What is your take on that?

- Which influences will play a role in parental participation, according to you?

- How do you think the expansion of screening will affect the confidence of parents and professionals in the heel prick test? How are confidence levels now?

- What do you think are some possible reasons that parents refrain from heel prick screening?

- Do you think that the **provision of information and counselling** about the heel prick screening should be revised as a result of this expansion, and how?

- Why / Why not? And if so, what should be added?
- Do you need more information or training? And what about colleagues / other professionals? Do you think they may require additional education? (*Do you see any problems / obstacles here?*)

**5. Profession-specific questions**

- Lab: how to deal with incidental findings that are not reported? What is your view of false positive results and false negative results and incidental findings?

- General practitioner: informing parents in case of abnormal results. How does this normally work? Do they have sufficient information (about process and content)? How do they view their own role?

- Paediatricians / metabolic doctors / paediatric neurologists: What are your experiences with current heel prick testing?

- Patient associations: What would the expansion of the heel prick screening mean for patient associations?
