# Supplementary material for: Expanding Neonatal Bloodspot Screening: A Multi-Stakeholder Perspective
Source: Front Pediatr. 2021 Oct 6;9:706394. doi: 10.3389/fped.2021.706394 (PMC8527172; doi:10.3389/fped.2021.706394)
Supplement: Supplementary file 2 [file Data_Sheet_2.docx]

**Supplementary file: *Expanding neonatal bloodspot screening: a multi-stakeholder perspective***

**Supplementary Appendix B. Interview Guide: Parents**

**1. Value of heel prick screening**

- How do you feel about your participation in the heel prick screening?
  - Did you know at the time what the purpose of the heel prick was?
  - To what extent did you feel it was a conscious choice to participate in the screening?
- If applicable:
  - What was your reason not to participate?
  - How was it receiving an abnormal result?

- What do you think are the important benefits of the heel prick?
- And what are important (or less important) disadvantages of the heel prick screening, in your opinion?

**2. Expansion of the heel prick screening: general**

- What do you think of the expansion of the heel prick screening?

**3. Ethics concerning the heel prick expansion**

*3.1 Screening of subgroups*

*This expansion also raises a number of new questions. For example, when testing for a particular disease, a distinction will be made between boys and girls. Boys with this disease are likely to suffer damage to the adrenal glands and brain. They can even die from the disease. When this disease is detected in boys in time, the disease can be well controlled. Girls with this disease develop other, often milder health complaints at a later age (40-60 years). The type that girls can get is not treatable. For that reason, girls will not be tested.*

- What do you think of this distinction between boys and girls in screening, and that girls who may have this disease are not tested?
- Do you think that parents should be informed of this when they receive the general information about the heel prick?

*3.2 (Untreatable) incidental findings*

*In some cases, there is an incidental finding. This means that the heel prick screening found something that we were not looking for. For example, it may come to light that the newborn infant has a****different****disease than the one the test was designed to detect.*

- Do you think that incidental findings should always be reported to the parents?
  - Is there a distinction between serious and non-serious illnesses, or between treatable and non-treatable diseases?
- Should the chance of such an incidental finding be reported in advance in the general information about the heel prick?

*3.3 Abnormalities in the mother*

*Sometimes, the heel prick screening in the child reveals that the mother, rather than the child, has a metabolic disease. Often mothers have no complaints of the condition at that time, and it is not clear whether they will ever suffer from it. Currently, mothers are referred to an internist by the paediatrician if there is evidence of illness in the mother. The internist then discusses with the mother if further investigation and possible treatment is necessary.*

- How do you feel about this?
- Do you think that mothers without complaints would want to know this?
  - Why / Why not?
- Should this be stated in advance with the general information?

*3.4 False positive results*

*As with almost every test, there is also a chance of a so-called 'false positive' result with the heel prick. In the case of a false positive result, the test initially seems to indicate signs of a disease in the newborn. Only after further investigation can parents be reassured: it was, as it were, a 'false alarm'. To give you an idea of ​​how often this happens: 170,000 children are given the heel prick every year. An abnormal result is found in just under 500 children. Of these 500, 200 turn out to have the disease and 300 do not.*

- What does this evoke in you?
- Does the chance of a false positive result affect your confidence in the heel prick?

**4. Information provision, influence on participation and trust**

- Do you think that the current expansion affects the willingness to participate in the heel prick screening, or does it influence confidence in the heel prick?

-  Do you think that parents need more or different information as a result of the expansion of the heel prick screening?

-  Do you think there should be limits to the heel prick screening?

- What do you think about having a choice of a 'plus package' that includes additional conditions (diseases) in the screening?

- - What kind of illnesses would qualify as far as you are concerned? *Untreatable disorders? Late-onset disorders?*
  - Does it matter to you whether genetic material (DNA) is tested directly? Why?

| **5. Background questions for the participant (to be completed after interview)** | |
| --- | --- |
| 1. What is your gender      2. What is your age?    3. What is your marital status? | □ Male  □ Female    …… years    □ Single  □ Married / Living together  □ Other, namely ……………. |
| 4. What is your highest level of education? (tick *one answer)* | □ None / Primary school  □ Pre-vocational secondary education  □ Secondary vocational education  □ Senior general secondary education  □ Pre university education  □ Higher professional education  □ University  □ Other, namely ………………………… |
| 5. To which population group do you consider yourself? | □ Dutch  □ Turkish  □ Moroccan  □ Surinamese  □ Antillean  □ Other, namely ……. |
| 6. To which religious/ideological group do you consider yourself?                  7. How many children do you have?      How many of them have had the heel | □ None  □ Roman Catholic  □ Protestant  □ Reformed  □ Islam  □ Jewish  □ Hindu  □ Buddhism  □ Other, namely …….     ____ (number) children, age (s): ­  ­­ _______ |
