# Supplementary material for: Expanding Neonatal Bloodspot Screening: A Multi-Stakeholder Perspective
Source: Front Pediatr. 2021 Oct 6;9:706394. doi: 10.3389/fped.2021.706394 (PMC8527172; doi:10.3389/fped.2021.706394)
Supplement: Supplementary file 3 [file Data_Sheet_3.docx]

**Supplementary file: *Expanding neonatal bloodspot screening: a multi-stakeholder perspective***

**Supplementary Appendix C: Code tree professionals’ interviews**

Acceptance

- No disadvantages
- Informed choice/undisputed choice
- Information provision
- False positives
- Expansion
- DNA test

Information provision:

- When and how much
- Acceptance
- Source and availability

Positive evaluation points (NBS):

- Follow-up
- Well organized
- High uptake

Aim of NBS

- Health gain

Points for improvement:

- Faster implementation of new disorders
- Follow up abnormal result.

Positive test result and follow-up

- Uncertainty about course of disease.
- Searching for information

Expansion: Unsolicited findings:

- Unsolicited findings: want to know the result when the professionals knows it
- Unsolicited findings: inform parents
- Unsolicited findings: not inform parents

Expansion:

- Acceptation
- Life planning
- Loss of golden life years/ right not to know
- Variable expression/penetrance
- Informed choice/undisputed choice
- Information provision
- Reproductive options
- Preventing diagnostic odyssey
- Untreatable unsolicited findings
- Untreatable disorders
- False positives
- Scientific research/participation in trials
- Diagnosing mothers with OCTN2

- Late-onset:
 - Treatability of the disorder
 - Protection of patient against too much information
 - Loss of golden life years/ right not to know

- Untreatable disorders:

- Definition of untreatability
 - Life planning
 - Preventing diagnostic odyssey
 - Reproductive options
- Loss of golden life years/ right not to know

- ALD:

- Health gain in boys
 - Feeling of unease about screening subgroups.
 - ALD: late onset/untreatability in girls
 - ALD: right not to know/open future/ loss of golden life years
 - ALD: reproductive choices
 - ALD: preparing girls for complaints
 - ALD: information provision

- OCTN 2:
 - inform parents
 - not inform parents

Future

- Additional package of optional disorders
- Limits screening
- NGS in NBS
- Therapy-driven expansion
