# Supplementary material for: Expanding Neonatal Bloodspot Screening: A Multi-Stakeholder Perspective
Source: Front Pediatr. 2021 Oct 6;9:706394. doi: 10.3389/fped.2021.706394 (PMC8527172; doi:10.3389/fped.2021.706394)
Supplement: Supplementary file 4 [file Data_Sheet_4.docx]

**Supplementary file: *Expanding neonatal bloodspot screening: a multi-stakeholder perspective*Supplementary Appendix D: Code tree parents’ interviews**

Acceptance

- False positives
- Information provision
- No disadvantages
- Informed choice/undisputed choice
- Expansion
- DNA test

Information provision:

- When and how much

Aim of NBS

- Health gain

Positive test result and follow-up

- Uncertainty about course of disease.
- Searching for information

Expansion:

- Life planning
- Loss of golden life years/ right not to know
- Variable expression/penetrance
- Want to know everything
- Informed choice
- Information provision
- Reproductive options

Expansion: Unsolicited findings:

- Unsolicited findings: want to know the result when the professionals knows it
- Unsolicited findings: inform parents
- Unsolicited findings: not inform parents

- Late-onset:
 - Treatability of the disorder
 - Protection of patient against too much information
 - Loss of golden life years/ right not to know

- Untreatable disorders:

- Definition of treatability
 - Life planning
 - Preventing diagnostic odyssey
 - Reproductive options
- Loss of golden life years/ right not to know

- ALD:

- Health gain in boys
 - Feeling of unease about screening subgroups.
 - ALD: late onset/untreatability in girls
 - ALD: right not to know/open future
 - ALD: reproductive choices
 - ALD: preparing girls for complaints
 - ALD: information provision

- OCTN 2:
 - inform parents
 - not inform parents

Future

- Additional package of optional disorders
- Limits screening
