# Supplementary material for: Expanding Neonatal Bloodspot Screening: A Multi-Stakeholder Perspective
Source: Front Pediatr. 2021 Oct 6;9:706394. doi: 10.3389/fped.2021.706394 (PMC8527172; doi:10.3389/fped.2021.706394)
Supplement: Supplementary file 5 [file Table_1.docx]

**Supplementary files: *Expanding neonatal bloodspot screening: a multi-stakeholder perspective***

**Table S1. Parent characteristics, n=17**

| Gender, *n (%)*  - Female  - Male | 15 (88.2)  2 (11.8) |
| --- | --- |
| Mean age, *years (range)* | 33.2 (27-40) |
| Marital status, *n (%)*  - Married/living together | 17 (100) |
| Educational level^a^, *n (%)*   - Medium - High | 4 (22.2)  13 (77.8) |
| Self-reported ethnicity b, *n (%)*  - Dutch | 17 (100) |
| Religion, *n (%)*   - None - Roman Catholic - Protestant - Reformed - Evangelical - Humanist | 9 (50)  3 (16.7)  2 (11.1)  1 (5.6)  1 (5.6)  1 (5.6) |
| Number of children, *mean (range)* | 2.3 (1-5) |

^a^Low: primary school, lower level of secondary school, lower vocational training; Medium: higher level of secondary school, intermediate vocational training; High: higher vocational training, university.
